# An Extensively Humanized Mouse Model to Predict Pathways of Drug Disposition and Drug/Drug Interactions, and to Facilitate Design of Clinical Trials[Fn FN3]

**DOI:** 10.1124/dmd.119.086397

**Published:** 2019-06

**Authors:** C.J. Henderson, Y. Kapelyukh, N. Scheer, A. Rode, AW. McLaren, A.K. MacLeod, D. Lin, J. Wright, L.A. Stanley, C.R. Wolf

**Affiliations:** Systems Medicine, School of Medicine, University of Dundee, Jacqui Wood Cancer Centre, Ninewells Hospital, Dundee, United Kingdom (C.J.H., Y.K., C.R.W., A.M., K.M., D.L.); Taconic Biosciences Inc., Rensselaer, New York (N.S., A.R.); Independent Consultant, Putley, Ledbury, Herts, United Kingdom (J.W.); and Independent Consultant, Linlithgow, West Lothian, United Kingdom (L.A.S.)

## Abstract

Species differences in drug metabolism and disposition can confound the extrapolation of in vivo PK data to man and also profoundly compromise drug efficacy studies owing to differences in pharmacokinetics, in metabolites produced (which are often pharmacologically active), and in differential activation of the transcription factors constitutive androstane receptor (CAR) and pregnane X receptor (PXR), which regulate the expression of such enzymes as P450s and drug transporters. These differences have gained additional importance as a consequence of the use of genetically modified mouse models for drug-efficacy testing and also patient-derived xenografts to predict individual patient responses to anticancer drugs. A number of humanized mouse models for cytochrome P450s, CAR, and PXR have been reported. However, the utility of these models has been compromised by the redundancy in P450 reactions across gene families, whereby the remaining murine P450s can metabolize the compounds being tested. To remove this confounding factor and create a mouse model that more closely reflects human pathways of drug disposition, we substituted 33 murine P450s from the major gene families involved in drug disposition, together with Car and Pxr, for human CAR, PXR, CYP1A1, CYP1A2, CYP2C9, CYP2D6, CYP3A4, and CYP3A7. We also created a mouse line in which 34 P450s were deleted from the mouse genome. Using model compounds and anticancer drugs, we demonstrated how these mouse lines can be applied to predict drug-drug interactions in patients and discuss here their potential application in the more informed design of clinical trials and the personalized treatment of cancer.

## Introduction

Data obtained during preclinical development often fail their extrapolation to the clinic; this is a major reason for the high rate of attrition in drug development (Kola and Landis, 2004; Kola, 2008). In addition, once a drug has reached the clinic, the capacity to improve drug treatment regimens by clinical trial is limited both by their expense and the numerous possible trial designs, particularly when drug combinations are being tested. Therefore, an urgent need exists to develop better in vivo models more predictive of human drug response (Kersten et al., 2017).

Studies in rodents play a pivotal role in elucidating human responses to drugs and environmental chemicals. They are also of critical importance in furthering our understanding of human disease by serving as models for drug efficacy testing and, more recently, as a model system for the personalization of the treatment of diseases such as cancer (Kim and Sharpless, 2012; Le Magnen et al., 2016). There are, however, some fundamental differences between rodents and humans in responses to drugs, including species differences in pathways of drug metabolism and disposition that can, at least in part, explain why preclinical studies frequently do not extrapolate to the clinic.

Four cytochrome P450 gene subfamilies—*Cyp1a, Cyp2c, Cyp2d* and *Cyp3a*—are predominantly involved in phase 1 drug metabolism in man (Rendic and Guengerich, 2015). In mice these comprise 34 P450s, whereas only five enzymes are responsible for the metabolism of the majority of drugs used clinically in man. In addition, the expression levels of many of these enzymes (which determine levels and variability in drug exposure) are controlled by two transcription factors, the constitutive androstane receptor (CAR) and the pregnane X receptor (PXR). These nuclear receptors also exhibit marked species differences in their activation by drugs and exogenous chemicals (Mackowiak et al., 2018).

To circumvent the species differences in drug disposition and to create models that more closely reflect human drug response, a number of humanized mouse lines have been reported. In some cases these have also involved humanization for both CAR and PXR (Gong et al., 2005; Dragin et al., 2007; Gonzalez, 2007; Ross et al., 2010; Scheer et al., 2010, 2012a,b, 2015; Scheer and Wolf, 2014; Durmus et al., 2015; Liu et al., 2015). These models have usually also involved the deletion of genes in the orthologous murine gene family. However, problems in the utility of these models have arisen in that cytochrome P450s in the remaining murine gene families can catalyze the reactions being studied. Indeed, compensatory changes resulting in the induced expression of murine P450s have been observed (van Waterschoot et al., 2008). In addition to this confounding factor, mice carrying individual humanizations are expensive to maintain, and any individual line would not allow for evaluation of the complexities of the possible interactions between multiple human P450s.

To create a model system that accurately reflects human pathways of phase 1 drug metabolism, we deleted 33 murine P450s of the *Cyp1a, Cyp2c, Cyp2d*, and *Cyp3a* gene subfamilies and replaced them with the major human drug metabolizing P450s CYP1A1, CYP1A2, CYP2C9, CYP2D6, CYP3A4, and CYP3A7, together with the transcription factors CAR and PXR. Through the use of this model the role of the P450 system in the disposition of a particular compound can be estimated, and we demonstrate how such models can be used to accurately predict human drug exposure and pharmacokinetics. We discuss their significant potential to improve the drug development paradigm and the design of combination clinical trials. We also discuss how the models can be applied, in an era of polypharmacy, to reduce the risks of serious drug-drug interactions and to personalize the treatment of disease.

## Materials and Methods

All chemicals were purchased from Sigma Aldrich (Poole, Dorset, UK) unless otherwise specified and were of the highest grade available.

### Animals

The animals used in this work were originally generated in a collaboration between CXR Biosciences (now Concept Life Sciences) and Taconic Biosciences in a project funded by the Scottish Government through the ITI program (principal investigators C.R.W. and N.S.). The mouse lines described in this manuscript were developed under a material transfer agreement between Taconic and the University of Dundee.

Taconic delivered cohorts of animals to University of Dundee and granted University of Dundee rights to breed, crossbreed, and maintain colonies of the animals at the Medical School Resource Unit, University of Dundee. All animals were supplied from the Medical School Resource Unit, University of Dundee, on a C57BL/NTac background. Mice were maintained in Tecniplast Sealsave micro-isolator cages containing Eco-Pure chip7D (Datesand Group, Manchester, UK) for bedding with ad libitum access to food (RM1; Special Diet Services, Stepfield, Essex, UK) and water, and a 12-hour light-dark environment. Temperature and relative humidity were maintained between 20°C and 24°C, and 45% and 65%, respectively.

All animal work described was approved by the Welfare and Ethical treatment of Animals Committee of the University of Dundee. Those carrying out this work did so with Personal and Project Licenses granted by the UK Home Office under the Animals (Scientific Procedures) Act 1986, as amended by EU Directive 2010/63/EU. Animals were inspected regularly by staff trained and experienced in small animal husbandry, with 24-hour access to veterinary advice.

At the end of studies, all animals were sacrificed according to Schedule 1 of the Animals (Scientific Procedures) Act 1986, by exposure to a rising concentration of CO_2_ and death confirmed by exsanguination.

Data in this paper was obtained using male and female mice at the ages specified in this section and elsewhere. Mice were age-matched to within 1 month of each other, unless otherwise stated. Animals were randomly assigned to control or treatment groups; analysts were not blinded to the identity of biologic samples.

Animal numbers were guided by power calculations (G*Power; www.gpower.hhu.de), pilot experiments, and previous experience, and experimental design was undertaken in line the 3Rs principles of replacement, reduction, and refinement (www.nc3rs.org.uk).

The 8HUM mouse line was generated by crossing a previously characterized multiple humanized model (Scheer et al., 2015) in which the *Cyp2c* (except *Cyp2c44*), *Cyp2d* or *Cyp3a* murine gene clusters and the transcription factors *Car* and *Pxr* were deleted, and replaced with the corresponding human genes (*CYP2C9*, *CYP2D6*, *CYP3A4*, *CYP3A7*, *CAR*, *PXR*) [Cyp2c^tm1897(albCYP2C9)Arte^ (Scheer et al., 2012a); Cyp2d^tm1836(CYP2D6*2)Arte^ (Scheer et al., 2012b); Cyp3a^tm1109(CYP3A4/3A7)Arte^-Cyp3a13^tm1728Arte^ (Hasegawa et al., 2011); Nr1i2^tm1198(NR1I2)Arte^ (Ross et al., 2010); Nr1i3^tm1089(NR1I3)Arte^ (Scheer et al., 2010)] with a line in which Cyp1a1 and Cyp1a2 were deleted and replaced with CYP1A1 and CYP1A2 (Cyp1a1^tm2025(CYP1A1)Arte^-Cyp1a2^tm2024(CYP1A2)Arte^) (Kapelyukh et al., manuscript in preparation). A total of 35 murine genes were replaced by eight human genes. Expression of human P450 genes was from the human promotor, except for *CYP2C9*, which was driven by the albumin promotor, and CYP1A1 and CYP1A2, which were driven by the murine *Cyp1a1* and *Cyp1a2* promoters, respectively. *CAR* and *PXR* were expressed off the corresponding murine promoters.

A model, CypC4KO, in which the *Cyp1a*, *Cyp2c*, *Cyp2d*, and *Cyp3a* gene clusters were deleted (34 genes, including *Cyp2c44*) but murine *Car* and *Pxr* were retained, was also generated.

8HUM mice were maintained by crossing males homozygous for the gene deletions and humanization described above with females heterozygous for the *Cyp2c* gene cluster deletion/*CYP2C9* humanization and retention of one allele of the murine *Cyp2c* gene cluster (see below). Homozygous CypC4KO mice were bred using a similar strategy.

### Animal Treatments

#### Rifampicin.

Mice were dosed intraperitoneally with rifampicin (RIF; 10 mg/kg in vehicle) or vehicle (corn oil) daily for 3 days. On day 4, mice were sacrificed for tissue collection.

#### 2,3,7,8-Tetrachlorodibenzodioxin.

Mice were given a single dose of 2,3,7,8-tetrachlorodibenzodioxin (TCDD) (10 *μ*g/kg) or vehicle (corn oil) by intraperitoneal injection, and samples of liver and small intestine were collected 48 hours after the dosing.

#### St. John’s Wort.

Mice were dosed with either St. John’s Wort (SJW) tablets (Kira LowMood relief St. John’s Wort extract 450 mg; batch XD092056, marketed in Germany as Jarsin; Klosterfrau Healthcare Group) or the active ingredient hyperforin (Phytoplan Diehm and Neuberger GmbH, Heidelberg, Germany).

An extract was made from the SJW tablets as follows: Tablets were crushed using mortar and pestle, mixed with 100% ethanol (1 ml per tablet), and placed on a rotator mixer (SB3; Stuart Scientific, UK) for 1 hour in a dark room. The mixture was centrifuged (16.1 krcf; 4°C; 10 minutes), and the supernatants were removed and pooled together in an amber Eppendorf tube. Just before administration the extract was mixed with PEG 400 (final ethanol concentration ∼13.9%) and given to mice at a volume of 5 ml/kg by oral gavage. The SJW extract dose was 312 mg/kg assuming 100% extraction efficiency by ethanol. Considering that measured hyperforin content in Jarsin (Kira) SJW extract is in a range of 2.22%–3.07% (Wurglics et al., 2001), the SJW extract dose we used in the study translates to 6.9–9.6 mg/kg of hyperforin. The dose of 18 mg/kg of hyperforin/adhyperforin dicycloammonium salt translates to 13.4 mg/kg of hyperforin/adhyperforin and to 10.7 mg/kg of hyperforin (the hyperforin content was ∼80% of total hyperforin/adhyperforin according to the reagent batch high-performance liquid chromatography analysis provided by the reagent supplier).

### In Vivo Pharmacokinetic Experiments

#### Pharmacokinetics of Caffeine, Debrisoquine, Midazolam, and Tolbutamide Administered as a Cassette Dose.

Female 8HUM mice received either two daily intraperitoneal doses of RIF (*n* = 4; 20–26 weeks) or vehicle (corn oil; *n* = 4; 25–29 weeks). On day three all animals were administered caffeine (5 mg/kg), debrisoquine (5 mg/kg), tolbutamide (5 mg/kg), and midazolam (3 mg/kg) as a cassette dose by oral gavage. Blood samples (10 *μ*l) were collected at the indicated time points after dosing, mixed immediately with 10 *μ*l of heparin (15 IU/ml), snap frozen in liquid nitrogen, and stored at approximately −70°C prior to analysis (see Supplemental Data for details).

#### Pharmacokinetics of Caffeine, Debrisoquine, and Midazolam Administered as a Cassette Dose to St. John’s Wort Pretreated Mice: Effect of Quinidine and Ketoconazole.

Two groups of female 8HUM mice received two daily oral doses of SJW (*n* = 6; 17–18 weeks) or vehicle (*n* = 6; 15–17 weeks; 13.9% ethanol in PEG400). On day three, one of the SJW-treated groups (*n* = 3) was administered ketoconazole (35 mg/kg) and quinidine (30 mg/kg) by oral gavage and the other two groups received vehicle (12% ethanol in PEG400) 30 minutes before administration of caffeine (5 mg/kg), debrisoquine (5 mg/kg), and midazolam (3 mg/kg) as a cassette dose by oral gavage. Blood sampling and analysis was carried out as described above and in Supplemental Data.

### Effect of Dabrafenib Pretreatment on Midazolam Pharmacokinetics

Female 8HUM mice received either two daily oral doses of dabrafenib (*n* = 3; 15 weeks; 10 mg/kg) or vehicle (*n* = 3; 16 weeks; 0.5% hydroxypropylmethylcellulose, 0.2% Tween 80). On day 3 both groups were administered midazolam (3 mg/kg) by oral gavage. Whole blood collection and sample preparation were performed as described above, and analytical details are described in Supplemental Data. A third group of female 8HUM mice (*n* = 3; 17–22 weeks) received two daily doses of dabrafenib (10 mg/kg) by oral gavage and were culled for tissue collection on day 3 to allow determination of the effects of dabrafenib on P450 expression at the start of the pharmacokinetic (PK) study.

### Effect of Sulfaphenazole on Pharmacokinetics of *S*-Acenocoumarol

Mice were administered two daily doses of SJW before receiving a single oral dose of *S*-acenocoumarol (1 mg/kg). *S*-acenocoumarol was dissolved in dimethyl sulfoxide (1 mg/ml) and diluted 10-fold in 100 mM potassium phosphate buffer (pH8) or in a solution of sulfaphenazole (9.78 mg/ml) in the same buffer. Compounds were administered to mice at a volume of 10 ml/kg. Blood collection and sample preparation was performed as described above and analytical details are provided in Supplemental Data.

### Effect of SJW and Hyperforin on Dabrafenib Pharmacokinetics

Mice were administered two daily doses of SJW, hyperforin/adhyperforin dicycloammonium salt (18 mg/kg), or vehicle (13.9 ethanol in PEG400) by oral gavage. On day 3 all animals were administered dabrafenib (10 mg/kg). Whole blood collection and sample preparation were performed as described above and analytical details are described in Supplemental Data.

### Pharmacokinetic Data Analysis

Pharmacokinetic parameters were calculated by Phoenix WinNonlin version 8.0.0.3176 (Certara, Princeton, NJ).

### Tissue Harvesting and Processing for Immunohistochemistry

Liver and duodenum were removed postmortem, rinsed in ice-cold sterile PBS, and fixed in Gurr buffer overnight before transfer into 70% (v/v) ethanol. The following day organs were dehydrated and embedded in paraffin and subsequently sectioned at a 5-*μ*m thickness using a Shandon Finesse 325 microtome. Sections were deparaffinized in xylene and rehydrated through a graded series of 100% to 50% (v/v) ethanol solutions, followed by immersion in distilled water. Antigen retrieval was carried out by immersion of slides in 10 mM sodium citrate buffer, pH 6, and heating in a microwave for 25 minutes on high power, before being allowed to cool to room temperature. After a washing in water, treatment for10 minutes with 0.3% hydrogen peroxide, and washing in phosphate-buffered saline (PBS) and PBS-Tween (0.05% Tween-20, v/v), slides were dried and a hydrophobic pen was used to draw round the section. Slides were then blocked [Dako Endogenous enzyme block, followed by a nonspecific block using normal goat serum (5% v/v) in PBS-Tween] before a washing in PBS-Tween and incubation overnight at 4°C with primary antibody [anti-rat CYP3A1 (Forrester et al., 1992)] 1:100 dilution in 5% normal goat serum in PBS-Tween. After a washing in PBS-Tween, slides were dried and incubated with secondary antibody (Dako anti-rabbit HRP) for 30 minutes at room temperature. Slides were washed in PBS-Tween and immunoreactivity visualized using Dako DAB+ chromogen kit according to manufacturer’s instructions. After a washing in PBS and water, slides were counterstained with hematoxylin before dehydration in a series of increasing-alcohol steps, ending in xylene. Slides were dried for 5–10 minutes before coverslips were mounted in DPX mountant.

Sections were examined using a Zeiss Axio Scope.A1 Polarized Light Microscope. Images of tissue sections were taken using a Carl Zeiss Axiocam color processed through Axiovision v4.8.2 imaging software.

### Preparation of Microsomes

Fresh liver samples from wild-type or humanized mice were homogenized in ice-cold SET buffer (0.25 M sucrose, 5 mM EDTA, and 20 mM Tris-HCL, pH 7.4) to make a 10% (w/v) homogenate solution (9 ml SET buffer/1 g liver) using Polytron PT-MR-2100 homogenizer. Liver homogenates were centrifuged at 2000 rpm (Sorvall RTH-250 rotor) for 10 minutes at 4°C. The supernatant was then spun at 12,000 rpm (Sorvall SS-34 rotor) for 20 minutes at 4°C. The resulting supernatant was centrifuged at 29,130 rpm (Sorvall TFT-45.6 rotor) for 90 minutes at 4°C, and the microsomal pellets were resuspended in ice-cold SET buffer and stored at −70°C.

Frozen individual duodenum samples were homogenized in SET buffer containing protease cocktail inhibitor (Roche) and phenylmethylsulfonyl fluoride (PMSF; 1 mM) using a Polytron homogenizer. Tissue homogenates were subjected to subcellular fractionation by differential centrifugation like that described for the preparation of liver microsomes. Microsomal fractions were stored at approximately −70°C prior to analysis. The protein concentration in the microsomal fractions was determined using a modification of the method of Lowry (Lowry et al., 1951) and bovine serum albumin standards, and total microsomal P450 was measured by difference spectra of reduced heme iron complex with carbon monoxide (Omura and Sato, 1964).

### Cytochrome c Reductase Activity

A mixture of cytochrome c from bovine heart (40 *μ*M) and mouse liver microsomes (∼7.6 *μ*g/ml) in 0.3 M potassium phosphate buffer (pH 7.7) was incubated at 37°C for at least 2 minutes before the reaction was started by addition of NADPH (0.1 mM final concentration). The absorbance at 550 nm was recorded and the reaction rate calculated from the linear region of the absorbance time course. The extinction coefficient of 0.0211 *μ*M^−1^cm^−1^ was used (van Gelder and Slater, 1962) to calculate concentration of the reduced cytochrome c.

### Dextromethorphan *O*-Demethylation

A mixture of dextromethorphan (10 *μ*M) and 8HUM liver microsomes (∼0.25 mg/ml protein) in phosphate buffer (100 mM KH_2_PO_4_ pH7.4, 3.3 mM MgCl_2_) was incubated for 5 minutes at 37°C prior to the addition of NADPH (final concentrations 1.3 mM). After 5 minutes, the reaction was stopped by combining 100 *μ*l of the reaction mixture with 120 *μ*l of 0.2 M hydrochloric acid containing dextrorphan-d3 (92 ng/ml) as an internal standard. The mixture was placed on ice for at least 20 minutes, centrifuged at 16.1 krcf for 15 minutes at +4°C, and 100 *μ*l of the supernatant transferred to a 96-well plate for analysis. The concentrations of dextromethorphan were measured by liquid chromatography–tandem mass spectrometry (LC-MS/MS). Chromatographic separation was performed on a Kinetex Biphenyl 100 Å column (5 *μ*m; 5.0 × 2.1 mm) (Phenomenex, Macclesfield, UK) using an injection volume of 10 *μ*l and a run time of 5 minutes. The detector used was a Micromass Quattro Micro mass spectrometer (Waters Corporation, Milford, MA) run in electrospray positive ion mode. The multiple reaction monitoring parameters for dextrorphan and dextrorphan-d3 were 258.16 and 261.24 (precursor ions) and 157.04 (one product ion for both compounds), respectively.

### In Vitro Kinetic Parameter Determination

All in vitro analyses were carried out in 100 mM potassium phosphate buffer, pH 7.4, containing 3.3 mM MgCl_2_, with agitation at 400 rpm at 37°C on a thermoshaker. All samples were handled in amber tubes under conditions of subdued light for the duration of the procedure. Incubations were initiated by the addition of NADPH to a final concentration of 1.3 mM and were terminated by transferring an aliquot of the reaction mixture, typically 50 *μ*l, to one volume of ice-cold acetonitrile containing 400 ng/ml of triazolam as internal standard, followed by vortexing (5 seconds), and incubation on ice. The samples were then centrifuged at 3000*g* for 55 minutes at 4°C and an aliquot of the supernatant fraction was analyzed by LC-MS/MS. Assays to determine the apparent kinetic parameters of 1-hydroxyl midazolam (CYP3A4), 4-hydroxyl tolbutamide (CYP2C9), and 4-hydroxyl debrisoquine (CYP2D6) formation in pooled rifampicin-treated male and female 8HUM mice (8HUM) or pooled human liver microsomes (HLM) were performed in triplicate under conditions of linearity for time (2, 40, and 40 minutes for 1-hydroxyl midazolam, 4-hydroxyl tolbutamide, and 4-hydroxyl debrisoquine, respectively) and microsome protein (0.05, 0.1, and 0.1 mg/ml for 1-hydroxyl midazolam, 4-hydroxyl tolbutamide and 4-hydroxyl debrisoquine, respectively).

Calibration curves were constructed with authentic metabolite standards and linear regression analysis used to calculate the amount of metabolite formed in each test sample. Apparent K_m_ and V_max_ values were estimated by nonlinear regression analysis (GraphPad Prism version 6.05) of the substrate concentration [S]-enzyme activity [V], fitting data to a Michaelis-Menten model. The 7-ethoxyresorufin *O*-deethylation assay was carried out as detailed below.

### 7-Methoxyresorufin *O*-Demethylation and 7-Ethoxyresorufin *O*-Deethylation

A mixture of either 7-methoxyresorufin or 7-ethoxyresorufin (1 *μ*M) and 8HUM liver microsomes or HLM (∼0.38 mg protein/ml) in 100 mM potassium phosphate buffer (pH 7.4) supplemented with MgCl_2_ (3.3 mM) was incubated at 37°C for 5 minutes before the addition of NADPH (final concentration 1.2 mM). Generation of the fluorescent product was registered in a kinetic mode using Fluoroscan Ascent FL (Labsystems; excitation filter 530 nm; emission filter 585 nm). Slopes of the linear part of the kinetic curves were calculated using Ascent Software Version 2.4.1 (Labsystems). For each reaction two controls, with and without reaction product resorufin (40 pmol) were run. Fluorescence was measured from both control wells and the difference in fluorescence intensity between wells with and without resorufin was used for the conversion of the relative fluorescence units to the picomoles of the reaction product.

### Dibenzylfluorescein *O*-Debenzylation

The *O*-debenzylation of dibenzylfluorescein (DBF) was measured as described above for the 7-methoxyresorufin *O*-demethylation assay, with the following modifications: the DBF concentration was 2 *μ*M; the excitation filter was 485 nm; the emission filter was 530 nm; 20 pmol of the fluorescein was added to the control wells.

### 7-Benzyloxyquinoline *O*-Debenzylation

The 7-benzyloxyquinoline debenzylation was measured as described above for the 7-methoxyresorufin *O*-demethylation assay, with the following modifications: The 7-benzyloxyquinoline concentration was 100 *μ*M; the excitation filter was 405 nm; the emission filter was 530 nm; 40 pmol of the metabolite 7-hydroxyquinidine was added to the control wells.

### Enzyme Inhibition Assay

Substrate cocktail containing caffeine (220 *μ*M), debrisoquine (67 *μ*M), tolbutamide(2 *μ*M), and midazolam (3 *μ*M) were incubated using 8HUM or HLM (UltraPoolHLM150, Mixed Gender, product no. 452117; Corning) (0.25 mg/ml) in the presence or absence of the isozyme-specific inhibitors fluconazole (300 *μ*M), sulfaphenazole (3 *μ*M), quinidine (3 *μ*M), paroxetine (10 *μ*M), or ketoconazole (1 *μ*M). After a 5-minute preincubation at 37°C, reactions were initiated by addition of NADPH (1.3 mM). In the case of furafylline (3 *μ*M), enoxacin (200 *μ*M), and paroxetine (10 *μ*M) the inhibitor was preincubated with NADPH (1.3 mM) in 100 mM potassium phosphate buffer (pH 7.4) and 3.3 mM MgCl_2_ for 30 minutes at 37°C prior to the addition of the substrate cocktail. After a 10-minute incubation (20 minutes for furafylline and enoxacin) the reaction mixture was quenched with 100 *μ*l of cold acetonitrile containing 0.3% formic acid and 400 ng/ml of triazolam as internal standard. The samples were then centrifuged at 3000*g* for 55 minutes at 4°C, and an aliquot of the supernatant fraction was analyzed by LC-MS/MS. All experiments were performed at least in triplicate.

### Microsomal Stability of *S*-Acenocoumarol

A mixture of acenocoumarol (1 *μ*M) and liver microsomes (2 mg protein/ml) from wild-type (WT), hepatic P450 reductase–null (HRN), Cyp2c KO, Cyp2c/Cyp2d/Cyp3a KO, or CYP2C9 humanized mice in phosphate buffer (100 mM KH_2_PO_4_ pH7.4, 3.3 mM MgCl_2_) was incubated in a water bath for 5 minutes at 37°C prior to the start of the reaction by addition of NADPH regenerating system (final concentrations: 1.3 mM NADPH, 4 mM glucose-6-phosphate, and 2 IU/ml glucose-6-phosphate dehydrogenase). The reaction mixture aliquots were taken at specified time points and mixed with an equal volume of acetonitrile containing warfarin (50 ng/ml) as an internal standard. The mixture was placed on ice for at least 20 minutes, centrifuged at 16.1 krcf for 15 minutes at +4°C, and 100 *μ*l of the supernatant was transferred to a 96-well plate for analysis. The concentrations of *S*-acenocoumarol were measured by LC-MS/MS. Chromatographic separation was performed on a Kinetex C18 100 Å column (2.6 *μ*m; 5.0 × 2.1 mm) (Phenomenex) using an injection volume of 10 *μ*l and a run time of 7 minutes. The detector used was a Micromass Quattro Micro mass spectrometer (Waters Corporation) run in electrospray positive ion mode. The multiple reaction monitoring parameters for *S*-acenocoumarol and warfarin were 354.06 and 309.1 (parent ions) and 163.08 and 251.15 (collision ions), respectively.

### Liquid Chromatography-Mass Spectrometry-Multiple Reaction Monitoring Analysis

Drug analyses of the in vitro incubations and in vivo PK samples were carried out by UPLC-MS/MS using a Waters Acquity UPLC (Micromass, Manchester, UK) and Micromass Quattro Premier mass spectrometer (Micromass) with electrospray detection. Chromatography was performed using a C18 column (Kinetex 1.7 *μ*m 100 A; 50 × 2.1 mm; Phenomenex) at a temperature of 45°C with mobile phases of 0.1% formic acid (A) and acetonitrile containing 0.1% formic acid (B). A gradient at a flow rate of 0.5 ml/min was run over 3 minutes as follows: 0–0.5 minutes: 95% A; 0.5–0.75 minutes: 95%–80% A; 0.75–1.50 minutes 80% A, 1.50–2.00 minutes 80%–50% A, 2.00–2.50 minutes 50%–5% A, then returning to initial conditions for final 0.5 minutes. The cone voltage and collision energy were optimized for each substrate and multiple reaction monitoring data were acquired (Supplemental Table 2).

## Results

**Fig. 1.**
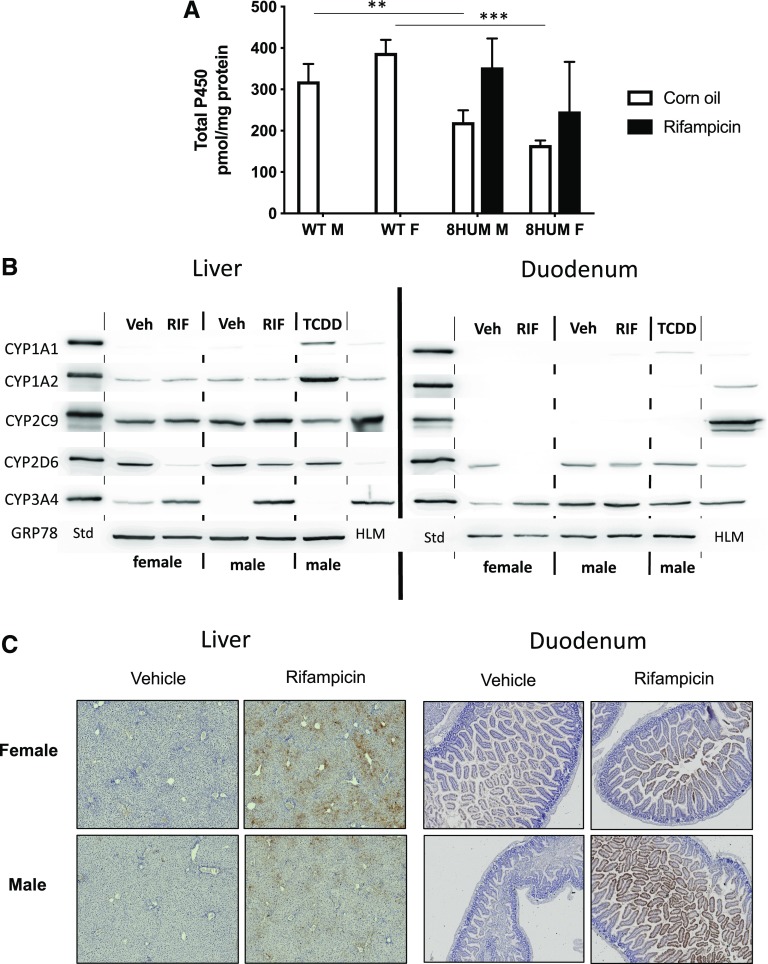
Expression of human cytochrome P450s in 8HUM mice. (A) Total hepatic P450 was determined from the carbon monoxide difference spectra (as described in *Materials and Methods*) in untreated male and female wild-type mice (WT; *n* = 3–8), and male and female 8HUM mice (*n* = 3) vehicle (white bars) and rifampicin-treated daily for 3 days (black bars; 10 mg/kg, i.p.). Data shown are mean ± S.D.; *t* test (two-tailed *P* values), statistically significant **P < 0.01; ***P < 0.001. (B) Immunoblot analysis of human P450 expression in liver and duodenum. Western blots were carried out on pooled microsomal fractions (*n* = 3) of liver and duodenum, as described in *Materials and Methods*, using antibodies specific for the human P450s shown. Std, His-tagged P450 standard; TCDD, 2,3,7,8-tetrachlordibenzodioxin-treated; Veh, vehicle-treated. (C) Immunohistochemical localization of CYP3A4 in liver and duodenum of vehicle- and rifampicin-treated 8HUM mice. Liver and duodenum from adult female 8HUM mice—vehicle- or rifampicin-treated for 3 days at 10 mg/kg i.p.—were processed for immunohistochemical staining as described in *Materials and Methods*. Photomicrographs shown are representative of *n* = 3.

The generation of the 8HUM and CypC4KO models together with the initial characterization of the 8HUM model are presented in the Supplemental Data. Total hepatic P450 in untreated 8HUM mice (male: 220 ± 29, female 165 ± 11 pmol P450/mg protein) were lower than those measured in wild-type animals (male 319 ± 42, female 388 ± 32 pmol P450/mg protein, *P* < 0.005 and *P* < 0.003, respectively, Fig. 1A). Following treatment with the PXR activator rifampicin P450 levels increased to 353 ± 70 (male) and 247 ± 120 pmol (female) P450/mg protein. The expression of individual hepatic P450 isozymes following RIF treatment was remarkably similar to the mean levels found in human liver (Fig. 1B). As the only human enzyme induced by RIF was CYP3A4, the change in total P450 content could be ascribed predominantly to the change in the level of this protein. Thus, of the total P450, CYP3A4 was 38% and 33% for males and females, respectively. This is very similar to the average level of this enzyme found in human liver (Michaels and Wang, 2014). As in humans and consistent with our previous findings (Hasegawa et al., 2011), CYP3A4 was expressed constitutively in the duodenums of 8HUM mice and was slightly induced on RIF treatment (Fig. 1B). CYP3A4 expression was also detected at lower levels in the jejunum and in the ileum (Supplemental Fig. 3). CYP2D6 was also expressed in this tissue at significant levels.

CYP3A4 was constitutively localized in a few cells around the central vein in the liver. Following RIF treatment, the staining became more intense and remained in this region, which is consistent with the localization of CYP3A4 in human liver (Palmer et al., 1992). In the small intestine, CYP3A4 was localized in the villi, the most intense staining being observed in the duodenum (Fig. 1C), which is also consistent with the localization of this enzyme in the human gastrointestinal tract (Fritz et al., 2019).

### 

#### In Vitro P450 Activity Measurements.

The in vitro hepatic activity of the human P450 enzymes expressed in the 8HUM model was determined by measuring the metabolism of a series of drugs that are substrates for specific human P450 enzymes (Table 1). Mice were pretreated with RIF to increase the expression of CYP3A4 (Fig. 1). The K_m_ value obtained for each human P450 was not significantly different (F-test) from that measured in pooled HLM (Table 1), inferring similar affinities in the 8HUM and HLM, allowing direct extrapolation of drug-drug interaction data obtained in 8HUM mice to man. The V_max_ values, which are determined by the level of expression of each individual isozyme, were also similar but in some cases higher than the HLM values.

**TABLE 1:** Kinetic parameters for probe compound metabolism in 8HUM mice Hepatic microsomal fractions from pooled rifampicin-treated male and female 8HUM mice or pooled human liver microsomes were used. Data presented as mean ± S.D. on biologic triplicate samples. F-test comparing 8HUM and HLM data for each substrate showed no significant difference. Enzyme activities were determined as described in Materials and Methods.

Substrate	CYP1A2 7-Ethoxyresorufin		CYP2C9 Tolbutamide		CYP2D6 Debrisoquine		CYP3A4 Midazolam	
	HLM	8HUM	HLM	8HUM	HLM	8HUM	HLM	8HUM
K_m_ (*μ*M)	0.22 ± 0.04	0.35 ± 0.05	73 ± 16	137 ± 36	25 ± 7	57 ± 8	0.73 ± 0.20	1.2 ± 0.3
V_max_ (pmol/min per milligram protein)	12.3 ± 0.5	20.7 ± 0.9	2.5 ± 0.1	2.7 ± 0.2	25 ± 2	98 ± 6	1500 ± 100	5100 ± 500

We then established the capacity of P450 isozyme inhibitors to reduce the metabolism of these substrates and compared the level of inhibition to that measured in a pooled human liver microsomal preparation (Table 2). A close correlation was observed between the level of inhibition measured with 8HUM samples and those with HLM. Metabolism of CYP1A2, CYP2C9, and CYP3A4 isoform-specific substrates in HLM and liver microsomes from 8HUM mice demonstrated a similar degree of inhibition by the isoform-specific inhibitors furafylline, enoxacin (CYP1A2), sulfaphenazole (CYP2C9), and ketoconazole (CYP3A4). Fluconazole inhibited metabolism of tolbutamide and midazolam to the same extent in both species, consistent with dual P450 isoform specificity of this inhibitor (Brown et al., 2006). Although the CYP2D6-specific reaction of debrisoquine 4-hydroxylation was inhibited in both HLM and microsomes from 8HUM mice by quinidine and paroxetine, the extent of the inhibition demonstrated some species differences (2- and 4-fold, respectively).

**TABLE 2:** In vitro inhibition of hepatic CYP1A2, CYP2C9, CYP2D6, and CYP3A4 in 8HUM and HLM microsomal fractions Data are presented as percent of activity measured in the absence of inhibitor. Values are mean ± S.D. from a minimum of 3 determinations. Other experimental details are given in Materials and Methods.

Inhibitors	Caffeine *(Paraxanthine)*		Debrisoquine *(4-OH Debrisoquine)*		Tolbutamide *(OH-Tolbutamide)*		Midazolam *(1-OH-Midazolam)*	
	HLM	8HUM	HLM	8HUM	HLM	8HUM	HLM	8HUM
Furafylline (CYP1A2)	53 ± 6*	56 ± 13*	65 ± 9	91 ± 13	103 ± 25	63 ± 19	95 ± 8	92 ± 7
Enoxacin (CYP1A2)	58 ± 15*	52 ± 8*	82 ± 29	89 ± 13	72 ± 27	73 ± 13	99 ± 13	90 ± 4
Fluconazole (CYP2C9/CYP3A4)	114 ± 37	100 ± 31	96 ± 9	88 ± 19	35 ± 21*	41 ± 33*	29 ± 4***	42 ± 5***
Sulfaphenazole (CYP2C9)	78 ± 26	67 ± 15	79 ± 25	88 ± 36	14 ± 4**	28 ± 15**	107 ± 21	125 ± 41
Quinidine (CYP2D6)	105 ± 28	74 ± 7	22 ± 5*	43 ± 5*	75 ± 17	132 ± 29	83 ± 9	97 ± 18
Paroxetine (CYP2D6)	66 ± 26	95 ± 22	52 ± 11*	13 ± 5**	113 ± 50	117 ± 34	91 ± 8	109 ± 17
Ketoconazole (CYP3A4)	135 ± 45	85 ± 34	142 ± 42	152 ± 11	154 ± 66	92 ± 11	6 ± 2***	11 ± 2***

* *P* < 0.05, **P < 0.01, ***P < 0.001, compared with no inhibitor; unpaired *t* test, two-tailed *P* values;

#### Effect of Rifampicin Treatment on Drug Pharmacokinetics.

The P450 enzymes provide an adaptive response to chemical challenge where the level of expression of individual enzymes is regulated by drugs, herbal medicines, industrial chemicals, and environmental agents in a manner that increases the rate of drug disposition. Pathways leading to the induction of the P450s, for example through CAR and PXR, are complex particularly when multiple drug combinations are being used. Currently available in vivo and in vitro models only give limited information about such interactions. P450 induction can result in loss of drug efficacy both for the primary target and the other drugs given concomitantly. Such effects can also increase the rate of formation of toxic metabolites.

**Fig. 2.**
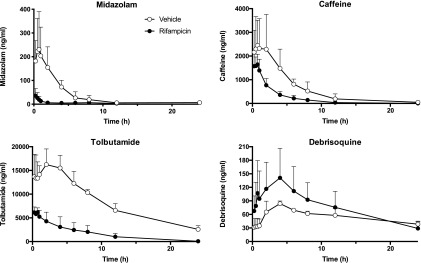
Effect of rifampicin on the pharmacokinetics of human P450 substrates. Adult female 8HUM mice (*n* = 3 or 4) were treated intraperitoneally with either vehicle (corn-oil; open circles) or rifampicin (closed circles) at 10 mg/kg, daily for 2 days, and on day 3 were administered by oral gavage a cassette comprising midazolam, caffeine, tolbutamide, and debrisoquine, and PK profiling was carried out as described in *Materials and Methods*. Data shown are mean ± S.D.

We carried out an in vivo study to investigate the effects of RIF pretreatment on the pharmacokinetics of the probe drug substrates (Fig. 2). In the case of the CYP3A4 substrate midazolam, pretreatment of mice with RIF markedly altered the pharmacokinetics of this compound, reducing drug exposure from an AUC_all_ of 860.3 ± 138.1 hour*ng/ml to 68.82 ± 21.14 hour*ng/ml (*n* = 4) vs. *P* = 0.001). Unexpectedly, RIF treatment also affected the clearance of compounds for which CYP3A4 is not the primary route of elimination, with the exposure of both caffeine (CYP1A2; AUC_all_ 5142 ± 685.1 hour*ng/ml vs. 14,574 ± 4253 hour*ng/ml; *P* = 0.05) and tolbutamide (CYP2C9; AUC_all_ 38,476 ± 10,218 hour*ng/ml vs. 197,956 ± 4157 hour*ng/ml; *P* ≤ 0.001) also being decreased. In contrast, debrisoquine exposure (CYP2D6; AUC_all_ 1860 ± 426.5 hour*ng/ml vs. 1331 ± 53.5 hour*ng/ml; n/s) was increased in response to RIF, possibly owing to the suppression of CYP2D6 by RIF; however, this difference was not significant.

To establish whether the reductions in drug exposure following RIF treatment were the result of changes in P450 enzyme activity, we measured in vitro rates of metabolism with probe substrates. The hydroxylation of 7-BQ, a CYP3A4 substrate, was increased approximately 2-fold as a consequence of rifampicin treatment (4218 ± 550 vs. 1889 ± 536 pmol/min per milligram; *P* = 0.03). There was no difference between the samples in metabolism of the CYP1A2 substrate 7-methoxyresorufin (42.84 ± 9.93 vs. 47.45 ± 3.65 pmol/min per milligram; not statistically significant). Incubations in the presence of the CYP1A2 inhibitor *α*-naphthoflavone (3.33 *μ*M) inhibited this activity in both sets of samples to a similar extent, 75% and 93%, respectively. The observed increase in caffeine clearance in vivo, was therefore not the result of changes in CYP1A2 activity. Tolbutamide 4-hydroxylation in vitro was also similar between rifampicin-treated groups and controls (71.22 ± 10.23 vs. 52.25 ± 7.17 pmol/min per milligram; n/s), and the CYP2C9 inhibitor sulfaphenazole (50 *μ*M) almost completely inhibited this activity in both groups (remaining activity 11.43 ± 9.38 vs. 2.01 ± 0.33 pmol/min per milligram; n/s). The reason for the unexpected changes in pharmacokinetics for caffeine and tolbutamide in this experiment are therefore intriguing and could be attributable to changes in phase 2 enzymes or to the expression of drug transporters.

**Fig. 3.**
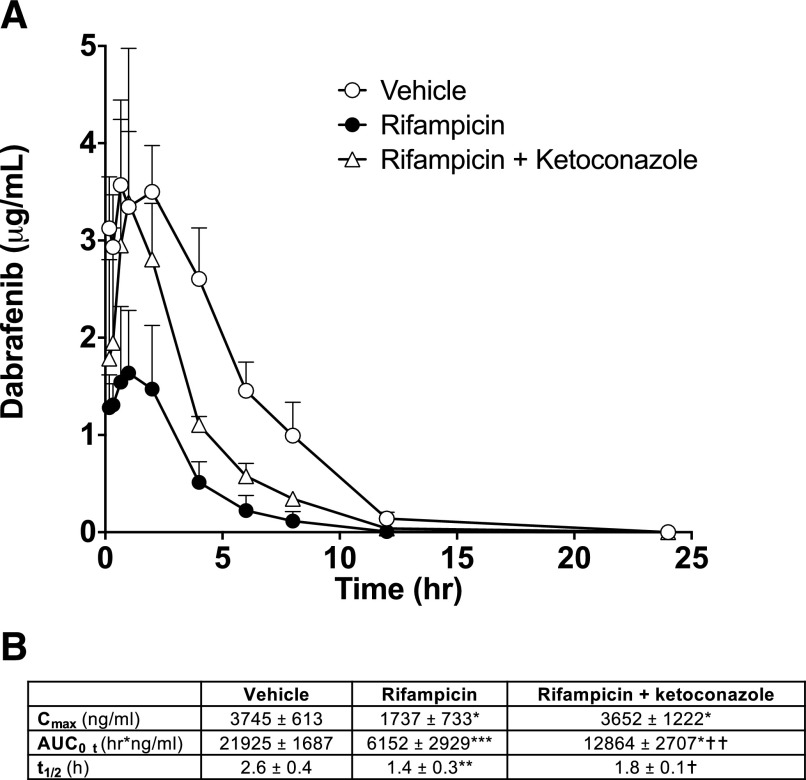
Effect of rifampicin and ketoconazole on dabrafenib pharmacokinetics. (A) Adult female 8HUM mice (*n* = 3) were treated with vehicle (corn oil, open circles) or rifampicin (10 mg/kg, closed circles) intraperitoneally daily for 3 days, with one of the latter groups receiving a dose of the CYP3A4 inhibitor ketoconazole (20 mg/kg PO, open triangles) 30 minutes before all groups were administered dabrafenib (10 mg/kg, PO), and PK profiling was carried out as described in *Materials and Methods*. (B) Pharmacokinetic parameters from the profiles shown in (A). Data shown are mean ± S.D. *P ≤ 0.05; **P ≤ 0.01; ***P ≤ 0.001 for vehicle vs. rifampicin and rifampicin vs. rifampicin + ketoconazole, *t* test. †*P* ≤ 0.05; ††*P* ≤ 0.01; for Vehicle vs. rifampicin + ketoconazole, *t* test.

We then investigated the effects of PXR activation on the pharmacokinetics of the anticancer drug dabrafenib, used as the standard of care in the UK for the treatment of BRAF mutant metastatic melanoma (Long et al., 2016). Female 8HUM mice were treated with vehicle (corn oil) or rifampicin (10 mg/kg, i.p) on three consecutive days prior to administration of dabrafenib [10 mg/kg, by mouth (PO)]. One group also received the CYP3A4 inhibitor ketoconazole (20 mg/kg, PO) 30 min before the administration of dabrafenib. Pretreatment with RIF markedly reduced the AUC of dabrafenib to approximately 28% of that in vehicle-treated animals; corresponding reductions in C_max_ and half-life were also observed (Fig. 3). Prior administration of ketoconazole significantly increased dabrafenib exposure, approximately doubling both the C_max_ and AUC (Fig. 3).

#### Detection of Human-Specific Metabolites in the 8HUM Mouse.

**Fig. 4.**
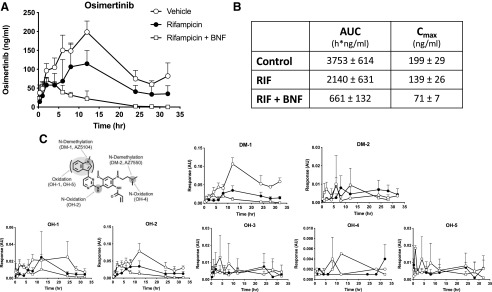
Effect of rifampicin and *β*-naphthoflavone on osimertinib pharmacokinetics. Adult female 8HUM mice (*n* = 3) were fed either normal RM1 diet (open circles), RM1 diet with rifampicin (RIF; equivalent dose 10 mg/kg, closed circles) for 3 days or RM1 diet with rifampicin (equivalent dose 10 mg/kg) and administered *β*-naphthoflavone intraperitoneally (BNF; 80 mg/kg, open squares) daily for 3 days, and on day 4 were administered osimertinib by oral gavage at a dose of 25 mg/kg, and PK profiling of osimertinib (A) and metabolites (B) was carried out as described in *Materials and Methods*. Osimertinib structure and sites of metabolism in humans (C) are also shown. Data shown are mean ± S.D.

Humans and rodents metabolize drugs down different pathways and also generate different metabolites both in their site of oxidation as well as in different metabolite ratios when multiple sites of oxidation are involved. The in vivo significance of this species difference can be of particular importance when the metabolites are either pharmacologically active, i.e., in drug efficacy, or toxic. To demonstrate the potential utility of the 8HUM mouse to recreate the bona fide pathways of human drug disposition we studied the metabolism of the epidermal growth factor receptor inhibitor osimertinib. Mice were pretreated with either RIF or a combination of RIF and *β*-naphthoflavone (BNF), an aryl hydrocarbon receptor agonist that induces P450s in the CYP1A gene family. The half-life of osimertinib in the 8HUM was greater than 20 hours versus 3.5 hours in WT mice [(Cross et al., 2014); Fig. 4]. The clearance of osimertinib was increased in 8HUM mice treated with RIF, and a further increase occurred when mice were also pretreated with BNF (Fig. 4). These data, consistent with our previous report using a simpler humanized model (MacLeod et al., 2018), suggest that members of the CYP1A gene family are involved in osimertinib disposition. The major human metabolites of osimertinib were detected in 8HUM mice (Fig. 4C). The above data together with our previous report demonstrate major species differences in the disposition of this compound both in the levels of drug exposure, in PK parameters, in the ratio of metabolites produced, as well as the P450 gene families involved (see *Discussion*).

#### Effects of Alternative PXR Activators on CYP3A4 Levels in the 8HUM Mouse.

RIF, although a potent human PXR activator, was toxic in the 8HUM model. On RIF treatment, liver enlargement and elevations in circulating liver enzymes were observed. On histopathological examination there was evidence of hepatic endothelial cell proliferation and increased numbers of Kupffer cells, suggesting possible hepatocyte damage. Oval cell and bile duct proliferation were also in evidence. We therefore sought to identify a specific, clinically relevant PXR agonist that was nontoxic. A number of PXR activators were tested in the simpler CAR/PXR/CYP3A4 model (Hasegawa et al., 2011; MacLeod et al., 2015, 2018). These were: the herbal medicine St. John’s Wort (SJW), the anti-HIV drug efavirenz (EFV), the anticonvulsant phenobarbital (PB), and the glucocorticoid dexamethasone (DEX). With the exception of DEX, all the compounds tested were effective inducers of CYP3A4 (Supplemental Fig. 4). Of particular note was SJW, which specifically induced CYP3A4 in the absence of toxicity and with only minimal induction of Cyp2b10 expression. Subsequent studies were therefore undertaken using SJW to induce CYP3A4 expression in 8HUM mice.

**Fig. 5.**
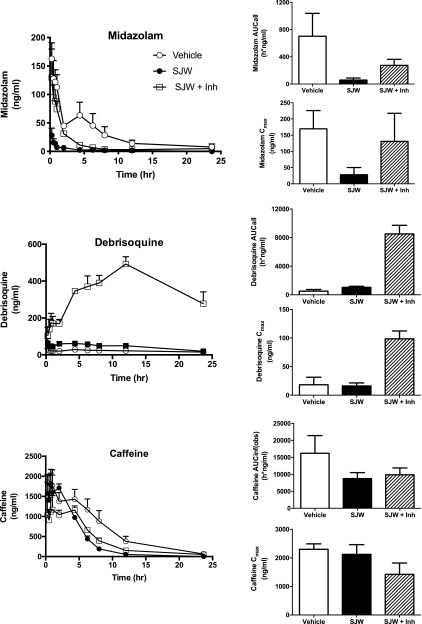
Pharmacokinetics of probe drugs in 8HUM mice treated with St. John’s Wort with and without P450 inhibitors. Female 8HUM mice were treated with vehicle (open circles, *n* = 5), St. John’s Wort (closed circles, *n* = 3), and SJW and ketoconazole and quinidine (open squares, SJW + inhibitor, *n* = 3) before being dosed with a drug cassette and a PK profile carried out as detailed in *Materials and Methods* for caffeine, debrisoquine, and midazolam. Data shown are mean ± S.D.

To test the effects of SJW as an inducing agent in vivo, animals received two daily doses of SJW, followed by a single cassette dose of midazolam, debrisoquine and caffeine. Two groups of mice also received a single dose of ketoconazole and quinidine 30 minutes prior to cassette administration. Treatment with SJW caused a 12-fold decrease in midazolam AUC, and additional administration of ketoconazole resulted in a 4.7-fold increase in exposure to the compound (Fig. 5; Supplemental Table 1). These data are consistent with induction of hepatic CYP3A4 and subsequent inhibition of the enzyme by the isoform-specific inhibitor. Treatment with SJW also resulted in a 2-fold increase in debrisoquine AUC, possibly owing to downregulation of constitutive CYP2D6 expression by PXR ligands in females (Fig. 1B) (Scheer et al., 2015). The CYP2D6-specific inhibitor quinidine caused an 8.1-fold increase in the AUC of debrisoquine in the SJW-treated group. Neither SJW nor ketoconazole or quinidine significantly affected the pharmacokinetics of the CYP1A2-specific substrate caffeine, although an approximately 50% reduction in AUC was observed on SJW treatment. The above data are similar to those obtained using RIF.

**Fig. 6.**
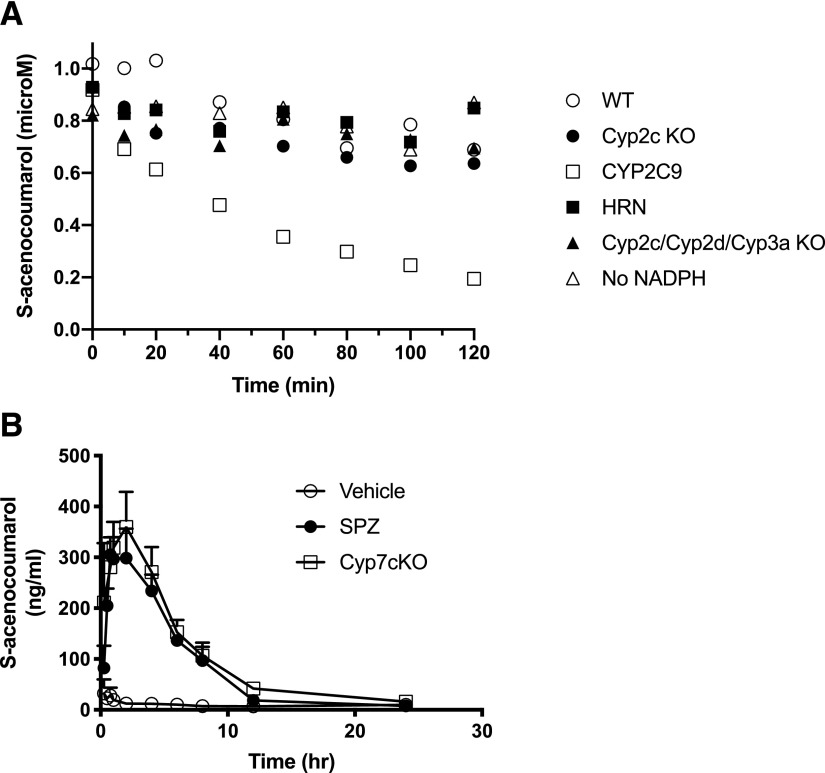
*S*-Acenocoumarol is a specific CYP2C9 probe drug in humanized mice. (A) In vitro microsomal stability assay. Liver microsomes from wild-type (open circles), CYP2C9 (open squares), Cyp2cKO (closed circles), Cyp2c/Cyp2d/Cyp3aKO (closed triangles), and hepatic P450 reductase null (HRN) (closed squares) mice were incubated with *S*-acenocoumarol and disappearance of drug monitored over time as described in *Materials and Methods*. Wild-type liver microsomes without NADPH (open triangles) were included as a control group. (B) Pharmacokinetics of *S*-acenocoumarol in 8HUM and CypC4KO mice. Female 8HUM and CypC4KO mice (*n* = 3) were treated with SJW and then administered *S*-acenocoumarol individually (open circles) or in combination with sulfaphenazole (closed circles) before PK profiling for *S*-acenocoumarol as described in *Materials and Methods*. 8HUM mice treated with SJW alone (open circles), 8HUM mice treated with SJW and sulfaphenazole (SPZ; closed circles), and CypC4KO mice were treated with SJW followed by *S*-acenocoumarol alone (open squares). Values show are mean ± S.D.

In view of the long half-life of tolbutamide, both in mice and humans, together with solubility issues and the indications that in addition to CYP2C9 other non-P450 pathways are involved in its disposition, we tested an alternative CYP2C9 probe drug, *S*-acenocoumarol (Ufer, 2005). *S*-acenocoumarol has several advantages over tolbutamide as it has a higher CYP2C9 specificity [fraction metabolized by CYP2C9 is 0.94 (Thijssen et al., 2001), compared with 0.85 for tolbutamide (Perkins et al., 2018)]. *S*-acenocoumarol is cleared rapidly in man (Thijssen et al., 2001), which is a distinct advantage in drug-drug interaction studies, where drug elimination is slowed down by the inhibitor and for accurate description of the terminal phase of the PK curve drug sampling is needed over a prolonged period. In addition, *S*-acenocoumarol is a rare example of a cytochrome P450 substrate that is resistant to metabolism by other murine NADPH-dependent enzymes. *S*-acenocoumarol metabolism in microsomes from wild-type, Cyp2cKO, Cyp2c/2d/3a KO, or hepatic P450 reductase–null (Henderson et al., 2003) mice was practically indistinguishable from that in the no cofactor control, whereas *S*-acenocoumarol was rapidly metabolized in liver microsomes from CYP2C9 humanized mice (Fig. 6A). In an in vivo drug-drug interaction study, 8HUM and CypC4KO mice were administered daily doses of SJW and on day 3 dosed with *S*-acenocoumarol, with one group of 8HUM mice coadministered with the CYP2C9 inhibitor sulfaphenazole (Fig. 6B). *S*-acenocoumarol AUC was increased 8.7-fold in 8HUM mice treated with sulfaphenazole compared with vehicle-treated mice (2008 ± 159 vs. 230 ± 30 hour*ng/ml), consistent with CYP2C9 inhibition in 8HUM mice by sulfaphenazole. The PK profile for *S*-acenocoumarol in the sulfaphenazole-treated 8HUM mice was essentially identical to that in CypC4KO mice (AUC 2520 ± 214 hour*ng/ml), suggesting almost complete inhibition of CYP2C9 in 8Hum mice (Fig. 6B).

**Fig. 7.**
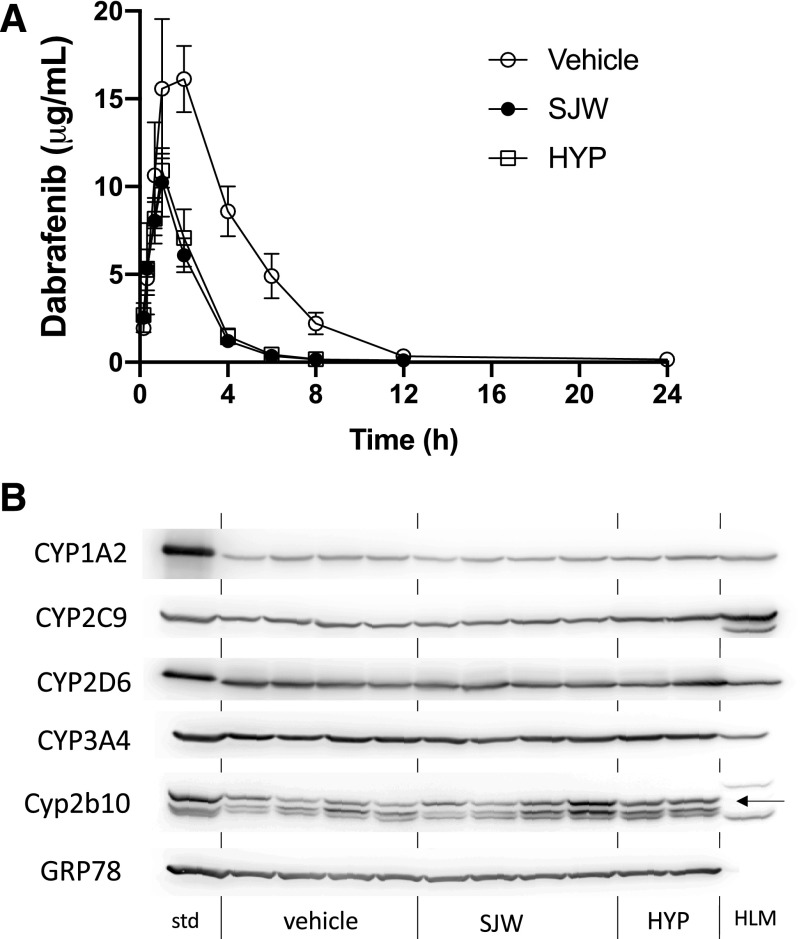
Effect of St. John’s Wort and hyperforin on pharmacokinetics. (A) Female 8HUM mice (*n* = 2–4) were treated with vehicle (open circles), St. John’s Wort (closed circles) or hyperforin (HYP; open squares) for 2 days as described before being dosed with dabrafenib for PK profiling as described in the *Materials and Methods*. Data are shown as mean ± S.D. (B) Liver microsomes from the 8HUM mice treated in (A) with vehicle, St. John’s Wort (SJW), or hyperforin (HYP), and also subject to dabrafenib PK profiling, were immunoblotted against CYP3A4 and Cyp2b10. Arrow indicates position of Cyp2b10 band. GRP78 was used as a loading control.

As SJW is a potent inducer of CYP3A4 expression in 8HUM mice at levels similar to human exposure, we investigated the effects of this preparation and the pharmacologically active constituent hyperforin on dabrafenib clearance. Dabrafenib pharmacokinetics were determined 2 days after treatment with these preparations. Treatment with either SJW or hyperforin decreased the AUC of dabrafenib by 69% and 65%, respectively (Fig. 7A; Table 3). Hepatic CYP3A4 levels in the animals from this study are shown in Fig. 7B. Interestingly, high levels of CYP3A4 expression were observed in animals not pretreated with either SJW or hyperforin, indicating that dabrafenib in its own right is a CYP3A4 inducer. This is consistent with the report that this compound is a CYP3A4 inducer in man (Lawrence et al., 2014; Puszkiel et al., 2019). Murine Cyp2b10 was also induced weakly by dabrafenib.

**TABLE 3:** Effect of St. John’s Wort and hyperforin on dabrafenib clearance in the 8HUM mouse Animal treatments and enzyme activity measurements were carried out as described in Materials and Methods. Data shown are mean ± S.D. from a minimum of three determinations.

	Vehicle	St. John’s Wort	Hyperforin
C_max_ (*μ*g/ml)	16.7 ± 2.9	10.2 ± 1.9**	10.9 ± 1
Terminal half-life (h)	1.9 ± 1.22	1.8 ± 0.9	1.2 ± 0.1
AUC_inf(obs)_ (h*μg/ml)	76 ± 3.2	24.2 ± 3.3****	26.7 ± 5.2***
CL/F_(obs)_ (ml/h per kilogram)	132 ± 5.5	419 ± 58****	383 ± 76**
Total hepatic P450 (pmol/mg protein)	286 ± 39	314 ± 80	276 ± 28
*n*	4	4	2

** *P* < 0.01, ***P < 0.001, ****P < 0.0001 for vehicle vs. SJW or hyperforin, *t* test.

**Fig. 8.**
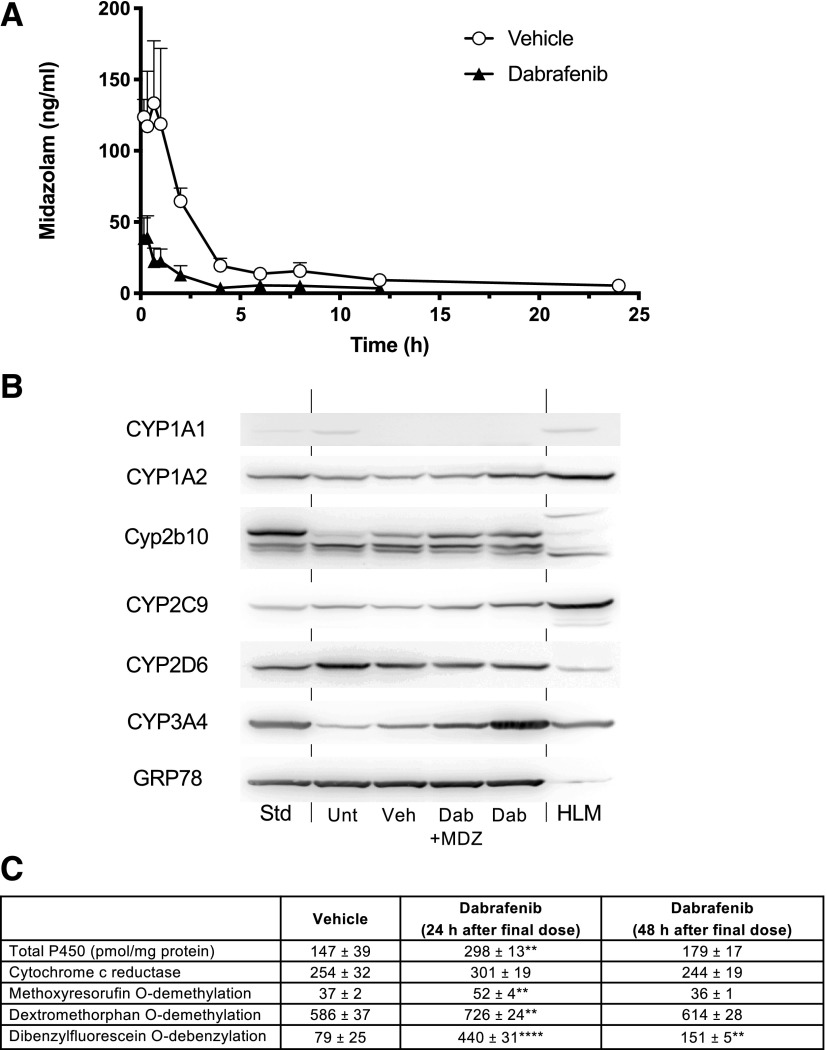
Effect of dabrafenib on midazolam pharmacokinetics in 8HUM mice. Adult female 8HUM mice (*n* = 3) were treated with vehicle or dabrafenib (10 mg/kg) PO daily for 2 days, and on day 3 were administered midazolam (3 mg/kg) PO and PK profiling carried out as described in *Materials and Methods*. (A) Pharmacokinetic profiling for midazolam in 8HUM mice receiving either vehicle (open circles) or dabrafenib (closed triangles). Data shown are mean ± S.D. (B) Immunoblotting of liver microsomes from 8HUM mice, untreated or treated as above, against mouse and human P450 isozymes. Dab, Dabrafenib; MDZ, midazolam; Unt, untreated; Veh, vehicle. One group of dabrafenib-treated mice (Dab) were sacrificed on day 3 without exposure to midazolam to determine the effects of dabrafenib on P450 expression at the start of the PK profiling. The other samples were collected after the completion of the PK study 24 hours later. Std, standard, recombinant human P450 protein or phenobarbital-treated liver microsomes (Cyp2b10); HLM, human liver microsomes. (C) Effects of dabrafenib treatment on hepatic cytochrome P450-related activities in liver microsomes from 8HUM mice 24 and 48 hours after drug treatment. Data shown are mean ± S.D. Unpaired *t* test (two tailed *P* values) statistically significant compared with vehicle **P ≤ 0.01; ****P ≤ 0.0001 for vehicle vs. dabrafenib at 24 or 48 hours, *t* test.

As the dose of dabrafenib used can be extrapolated to that used in patients, we investigated its capacity to alter the pharmacokinetics of the CYP3A4 substrate midazolam. Pretreatment with dabrafenib markedly increased midazolam clearance compared with vehicle-treated animals, AUC_all_ values decreasing to 91 ± 15 from 462 ± 70 hour*μg/ml, respectively (Fig. 8A).

Western immunoblot analysis of samples from this experiment showed induction of CYP3A4, which was most marked 24 hours after the final dose of dabrafenib (Dab vs. Dab + MDZ; Fig. 8B). No marked induction of the other human P450s in the 8HUM was observed, although some induction of murine Cyp2b10 was detected. Dabrafenib significantly increased total hepatic microsomal P450 levels, methyoxyresorufin *O*-demethylation, dextromethorphan *O*-demethylation, and 7-benylfluorescein *O*-debenzylation activities 24 hours after the last dabrafenib dose and at the start of the PK profiling (Fig. 8C). The most marked effect of dabrafenib was on 7-benylfluorescein *O*-debenzylation, which was increased approximately 5.5-fold 24 hours after the second dabrafenib dose and remained elevated (at about twice control values) 24 hours later.

#### Relation of Drug/Drug Interactions Observed in 8HUM Mice to Clinical Measurements.

We used the experimental data obtained using the 8HUM model to evaluate how closely it predicted clinically observed drug/drug interactions. In 8HUM mice a ketoconazole dose of 35 mg/kg is equivalent to a dose 200 mg/70 kg body weight in man, on the basis of the body surface area normalization method (Reagan-Shaw et al., 2008). In a healthy volunteer study, a single dose of 200 mg ketoconazole resulted in an ∼5-fold increase of midazolam AUC when given 2 hours prior to midazolam (McCrea et al., 1999). This was in close agreement with the 4.7-fold increase in midazolam AUC observed in 8HUM mice when the equivalent dose of ketoconazole was administered (Supplemental Table 1).

The interaction of debrisoquine with quinidine given as a single dose in man has not been reported. We therefore compared the inhibitory effect of quinidine on the PKs of debrisoquine in 8HUM to that of desipramine in man because both compounds have a similar fraction metabolized (f_m_) in the corresponding species (VandenBrink et al., 2012). Quinidine increased debrisoquine AUC 16.7-fold compared with vehicle-treated 8HUM mice. Although the AUC ratio was 8.1-fold higher compared with the SJW-treated group, this reduction was possibly the result of suppression of CYP2D6 expression by PXR ligands in the humanized mice (Scheer et al., 2015). The latter fold change in AUC corresponds to a CYP2D6 f_m_ of 0.88 for debrisoquine (eq. 1), assuming complete inhibition of CYP2D6 by the quinidine. In man desipramine has a CYP2D6 f_m_ of 0.88 (VandenBrink et al., 2012). Administration of 200 mg quinidine resulted in a 7.5-fold increase of desipramine AUC in man (Brøsen and Gram, 1989), which was extremely close to 8.1-fold increase of debrisoquine AUC in eight HUM mice after coadministration with 30 mg/kg quinidine. The 30-mg/kg quinidine dose in mice is equivalent to 170 mg/70 kg body weight man (Reagan-Shaw et al., 2008).(1)
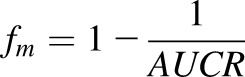
The interaction of *S*-acenocoumarol with sulfaphenazole in man has not been reported, so we used the effect of sulfaphenazole on tolbutamide pharmacokinetics (Veronese et al., 1990) to predict the *S*-acenocoumarol AUC increase in man. The tolbutamide AUCR (eq. 2) was related to sulfaphenazole concentration and tolbutamide fraction metabolized by CYP2C9 using the published reverse inhibition component of the “combined model” of drug-drug interaction (Fahmi et al., 2009).(2)
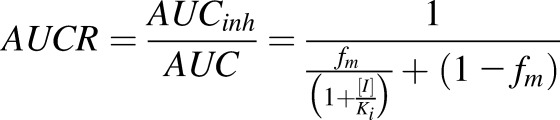
*AUCR* is the ratio of the drug area under the curve in the presence of inhibitor (*AUC_inh_*) to that without the inhibitor (*AUC*); [*I*] is the concentration of inhibitor; *K_i_* is the dissociation constant for enzyme-inhibitor complex; *f_m_* is the fraction of the drug metabolized by the inhibited enzyme. Equation 2 can be rearranged to:(3)
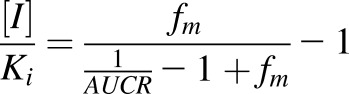
Perkins et al. (2018) estimated the fraction of tolbutamide metabolized by CYP2C9 to be 0.85 and the *AUCR* has been reported to be 5.3 (Veronese et al., 1990). Using the values reported in the above studies in eq. 3 gave an [*I*]/K value of 21 for sulfaphenazole. The fraction of *S*-acenocoumarol metabolized by CYP2C9 was calculated from the AUCR value of 16.3 for *S*-acenocoumarol in CYP2C9 extensive versus poor metabolizers (Thijssen et al., 2001) as shown in Eq. 4:(4)
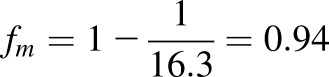
By applying the dose of sulfaphenazole used in the tolbutamide study (Veronese et al., 1990) to predict its effect on *S*-acenocoumarol AUC in man, we used the [*I*]*/K_i_* value of 21 in eq. 4 together with 0.94 as a fraction metabolized (*f_m_*).(5)
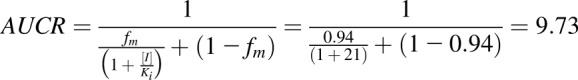
The predicted *AUCR* in man (9.73 from eq. 5) was close to that observed in 8HUM mice (8.72). It should be noted that 8HUM mice received a single dose of 88 mg/kg sulfaphenazole, equivalent to a single dose of 500 mg/70 kg human (Reagan-Shaw et al., 2008), whereas in the volunteer study sulfaphenazole was given every 12 hour at a dose 500 mg for 48 hours. However, semi–physiologically based pharmacokinetic modeling suggested that the inhibitory effect of sulfaphenazole given at multiple doses would not significantly differ from a single inhibitor administration of 500 mg, because of the very high inhibition potency and low elimination rate of this CYP2C9 inhibitor (Kanamitsu et al., 2000). Indeed, chronic administration of 1 g sulfaphenazole inhibited tolbutamide elimination to an extent like that of a single dose of this compound (Pond et al., 1977). Thus, multiple 500-mg doses of sulfaphenazole should produce a similar AUCR for *S*-acenocoumarol in man as a single dose, which allows comparison of the observed 8HUM data to that predicted in man.

## Discussion

The 8HUM model probably represents one of the most complex humanized transgenic models generated to date in that 35 murine genes were substituted for their human counterparts. We show that 8HUM mice can reflect many of the characteristics of human drug metabolism and disposition and accurately predict drug-drug interactions in man. The 8HUM line has significant advantages over previously humanized models in that the potential confounding effects of murine P450 enzymes, including compensatory changes in expression on drug disposition, have to a large degree been eliminated (van Waterschoot et al., 2008; Scheer et al., 2015; Bissig et al., 2018), and the relative importance of human P450s from different gene subfamilies in the disposition of a compound can be assessed in a single model, as shown here for osimertinib (MacLeod et al., 2018).

Some of the P450s, notably CYP3A4, were expressed off the human promoter, allowing the level of hepatic CYP3A4 to be regulated by the application of CAR or PXR activators and facilitating investigation into the relationship between the variability in CYP3A4 expression and drug efficacy or toxicity. CYP1A1 and CYP1A2 expression can also be regulated through the application of aryl hydrocarbon receptor activators such as BNF. We have recently shown that CYP1A1 is constitutively expressed at extremely low levels in all tissues of CYP1A1/1A2-humanized mice, which was one of the founder lines used to create the 8HUM line. CYP1A1 was significantly induced to high levels in the liver and lungs, and to a lesser degree in the gastrointestinal tract and kidney, of CYP1A1/1A2-humanized mice (manuscript in preparation). It is interesting that CYP3A4 was expressed constitutively in the gastrointestinal tract but not in the livers of males and at a low level in females. By comparing drug pharmacokinetics with and without pretreatment with a PXR activator and with and without a specific CYP3A4 inhibitor, the relative role of gastrointestinal versus hepatic drug metabolism in drug bioavailability can be established. We show that a number of drugs known clinically to induce CYP3A4 expression in patients also induce in the 8HUM model. It has been previously shown, using simpler humanized CAR/PXR models, that compounds such as RIF, CITCO, TCPOBOP, and dexamethasone exhibit profound species differences in the activation of these transcription factors. Such effects can confound the extrapolation of preclinical studies to man (Scheer et al., 2008, 2010). For example, we show here that dexamethasone, a potent murine activator of Car/Pxr at a dose of 10 mg/kg (Hasegawa et al., 2011), does not activate human CAR/PXR in 8HUM mice at this dose. The 8HUM can therefore be used to determine PK/PD relationships for CAR/PXR activation that can then be extrapolated to the clinical situation for single drugs or drug combinations. Also, drugs in development may not exhibit efficacy in a murine model owing to activation of murine Car or Pxr, which does not reflect what will happen in man [see Stanley et al. (2006)].

The antitubercular drug RIF has often been used as a specific potent activator of human PXR and is also a potent CYP3A4 inducer in patients. However, RIF was hepatotoxic in the 8HUM model, compromising its utility as a generic CYP3A4 inducer in pharmacokinetic and drug efficacy studies. Our finding that 8HUM mice are uniquely susceptible to the toxicity of RIF (no toxic effects were observed in WT mice even at much higher doses), is intriguing and merits further investigation to determine its relevance to man. However, it is known that human PXR modulates the hepatotoxicity associated with RIF and isoniazid cotherapy via a PXR-mediated alteration of heme biosynthesis; this has previously been studied in WT, Pxr-null, and hPXR mice (Li et al., 2013). Through the use of 8HUM mice such toxicities can be studied against a background that is not only humanized for PXR but also CAR and all the key human drug-metabolizing P450s. Furthermore, the early onset of toxicity in this model presents the possibility that, with further validation, the 8HUM mouse could find utility as a rapid onset model for the study of drug-induced liver injury.

The 8HUM model has numerous potential applications in the quest to improve drug therapy both in the development of new drugs and in improving prescribing practices for currently used drugs (Table 4). Recent studies have highlighted the dramatic increase in drug prescribing in general practice. In 2010 nearly 20% of individuals over the age of 70 take 10 or more drugs per day, excluding hospital, herbal, and over-the-counter medicines, with an extremely high risk of serious drug-drug interaction (Guthrie et al., 2015). It is impossible to test the potential consequences of these drug combinations by clinical trial, and the use of the 8HUM model provides a means to test hypotheses and predict clinical outcomes at an experimental level. This is also the case in the design of clinical trials, in which drug combinations are increasingly being used, and particularly so in the treatment of cancer, where the numerous possible drug combinations involving targeted antitumor agents [most of which are P450 substrates and as shown here also PXR agonists (Zanger and Schwab, 2013)] are being tested to increase efficacy and delay the onset of drug resistance.

**TABLE 4:** Applications of the 8HUM mouse

Define the role of cytochrome P450s in vivo drug/xenobiotic disposition (CypC4 KO)
Improve the predictive power of drug efficacy studies with more human-like PK and in vivo generation of human drug metabolites
More accurate predictions of individual patient responses to targeted anticancer drugs
Understanding how variability in P450 isozymes affects drug efficacy
Potential application in the study of drug-induced toxicity
Predicting potential drug-drug interactions for single and complex drug mixtures and how these could be circumvented
Studying the interaction of drugs with herbal medicines and environmental agents
Defining PK/PD relationships for chemopreventive agents
Improve in silico algorithms for extrapolating laboratory data to the clinic

Using the 8HUM model, drug exposures are obtained that are close to those observed in man. We also show that human metabolites are generated and that drug-drug interactions involving P450-P450 or P450-CAR/PXR can be evaluated. The model can therefore be applied to the more informed design of complex clinical trials in a manner that increases the probability of a positive outcome. Our studies with dabrafenib and osimertinib exemplify these points. For osimertinib the half-life is 3.5 hours in WT mice, 20 hours in 8HUM mice, and 55 hours in patients (Cross et al., 2014). All the known human metabolites are produced, and the major murine osimertinib metabolite [N-oxidation, OH-2 (MacLeod et al., 2018)] responsible for the rapid clearance in mice is produced at much lower levels in 8HUM mice. We have shown that dabrafenib at clinically relevant doses is a potent PXR activator and CYP3A4 inducer. The predicted clinical consequence is that it will induce its own metabolism, resulting in reduced exposure; indeed, reductions in the half-life (from 4.2 to 2.1 hours) and AUC_0__-t_ (from 9359 to 6545 hour*ng/ml) have been reported in patients whose blood levels of dabrafenib were measured on days 1 and 18 of treatment at 150 mg twice per day (Suttle et al., 2015). In addition, dabrafenib probably alters the metabolism of other CYP3A4 or MDR1 drugs used concomitantly, such as glucocorticoids.

In the light of RIF hepatotoxicity, we identified SJW as a potent, nontoxic PXR activator to use as a generic inducer of hepatic CYP3A4. Interestingly, the hyperforin content of our SJW extract (6.9–9.6 mg/kg; see *Materials and Methods*) was very like that of the hyperforin we used (10.7 mg/kg), and both significantly reduced dabrafenib exposure to similar extents, as reflected in several PK parameters, demonstrating the potential effect on circulating dabrafenib levels.

Although the prediction of human PK parameters from in vitro experiments has markedly decreased the number of new chemical entities failing clinical trials, preclinical methods still need improvement (Kola and Landis, 2004). The estimation of in vivo human clearance from in vitro data has a tendency to significantly under-predict (Hallifax and Houston, 2009; Hallifax et al., 2010; Wood et al., 2017), whereas the in vitro approach tends to over-predict the magnitude of drug-drug interactions in vivo (Vieira et al., 2014). Animal studies for the preclinical assessment of potential drug-drug interactions are hindered by significant species differences in metabolism and the consequential difference in fraction of drug metabolized. For example, for the human CYP2D6-specific substrate bufuralol, wild-type mice are rapid metabolizers, whereas for another CYP2D6-specific substrate, debrisoquine, wild-type mice have the profile of slow metabolizers (Scheer et al., 2012b). Humanization for individual cytochrome P450s or even for entire P450 clusters does not solve the problem of the effects of enzymes from other P450 subfamilies (Perloff et al., 2000; van Waterschoot et al., 2008). The 8HUM model has all major mouse cytochrome P450s replaced by their human orthologs and thus has the potential to be a tool with improved accuracy for prediction of drug-drug interaction in the preclinical stage of drug development. The issue of defining the fraction of drug metabolized for such calculations can be resolved through the use of the CypC4KO model as illustrated for *S*-acenocoumarol.

Over the last 30 years advances in our understanding of drug metabolism has markedly improved the drug development process and enhanced our understanding of drug-drug interactions. In this regard, a variety of informative in silico and laboratory-based model systems have been developed to predict drug exposure and drug-drug interactions in man with varying degrees of success. Researchers in industry depend increasingly on in silico approaches such as simulation tools and the SimCyp family of modeling; however, it is still recognized that the prediction of drug disposition from in vitro and in silico data needs fundamental improvement (Wood et al., 2017). Through the use of more complex humanized models, many of the shortcomings of simpler humanized models, e.g., redundancy between murine gene families, is avoided. However, it is acknowledged that, by definition, no model is perfect and that factors such as species differences in plasma protein binding and biliary elimination will not be addressed necessarily in a model such as the 8HUM, although with regard to the former an extensive study showed good correlation between protein binding in humans, rats, mice, and dogs for >500 drugs (Colclough et al., 2014).

With regard to the 8HUM model, it should be noted that a number of murine P450s remain, e.g., Cyp2a, Cyp2b, Cyp2e gene families; however, their roles in drug disposition can be evaluated using CypC4 KO and HRN mice. It is also worthy of note that other pathways in the 8HUM model have not been humanized, e.g., drug transporters such as Abcb1/b2, Abcg1; however, species differences in these transporters are poorly defined (Chu et al., 2013) and their role in drug disposition can be established using knockout animals whose transporters have been deleted or through the use of inhibitors whose specificity is enhanced when used in conjunction with the CypC4 KO model.

The sophisticated 8HUM model described here complements these approaches by providing a powerful approach for establishing the role of the human P450 system in drug disposition and defining the role of other pathways of drug elimination, such as those mediated by drug transporters and/or phase 2 enzymes. It has significant potential in predicting human responses to drugs, extrapolating drug efficacy studies to man, addressing cases where human metabolism differs markedly from that in conventional animal models, and personalizing the treatment of disease. To further improve the versatility of the model for drug efficacy studies and to establish PK/PD relationships, 8HUM versions of murine disease models could also be created (e.g., using CRISPR technology). Such models will be of value in determining how variability in drug exposure affects drug responses toxicities, facilitating studies into the personalization of drug treatment. In this regard, we have a particular interest in the use of the models for the personalized treatment of cancer to improve the predictive power of patient-derived xenograft drug sensitivity studies (Hidalgo et al., 2014). We are currently making an immunodeficient version of the 8HUM model for this purpose.

## Abbreviations

7BQ: 7-benzyloxyquinoline
BNF: *β*-naphthoflavone
CAR: constitutive androstane receptor
HLM: human liver microsome
HRN: hepatic P450 reductase–null
krcf: relative centrifugal force x1000
LC-MS/MS: liquid chromatography–tandem mass spectrometry
P450: cytochrome P450
PD: pharmacodynamic
PK: pharmacokinetic
PXR: pregnane X receptor
RIF: rifampicin
SJW: St. John’s Wort
WT: wild type
